# Integrated prenatal and postnatal management for neonates with transposition of the great arteries: thirteen-year experience at a single center

**DOI:** 10.1186/s13052-024-01730-w

**Published:** 2024-08-22

**Authors:** Xieyi Lin, Ying Huang, Wen Xie, Lu Chen, Yuping Huang, Yu Huang, Bingyu Ma, Shusheng Wen, Wei Pan

**Affiliations:** 1 Department of Cardiovascular Pediatrics, Guangdong Cardiovascular Institute, Guangdong Provincial People’s Hospital, Guangdong Academy of Medical Sciences, Guangzhou, 510080 P.R. China; 2 Department of Cardiovascular Surgery, Guangdong Cardiovascular Institute, Guangdong Provincial People’s Hospital, Guangdong Academy of Medical Sciences, Guangzhou, 510080 P.R. China; 3 Department of Endocrinology and Metabolism, Peking University Third Hospital, Beijing, China; 4 Department of Cardiovascular Surgery, Guangdong Cardiovascular Institute, Guangdong Provincial People’s Hospital, Guangdong Academy of Medical Sciences, Southern Medical University, Guangzhou, 510080 P.R. China; 5 Department of Cardiac Maternal-Fetal Medicine, Guangdong Cardiovascular Institute, Guangdong Provincial People’s Hospital, Guangdong Academy of Medical Sciences, Southern Medical University, Guangzhou, 510080 P.R. China

**Keywords:** Transposition of the great arteries, Prenatal diagnosis, Postnatal outcome, Arterial switch operation

## Abstract

**Background:**

Transposition of the great arteries (TGA) is the most common cyanotic congenital heart defect in neonates but with low prenatal detection rate. This study sought to review the prenatal diagnosis, associated abnormalities, and mid-term postnatal outcomes of fetuses with TGA and investigate the integrated prenatal and postnatal management for TGA neonates.

**Methods:**

A total of 134 infants prenatally diagnosed with TGA in Guangdong Provincial People’s Hospital, China, from January 2009 to December 2022 were included in the study. The prenatal ultrasound data and neonatal records were reviewed to assess the accuracy of prenatal diagnosis. Univariate and multivariate logistic and Cox analyses were used to identify risk factors associated with prognosis in such individuals.

**Results:**

The population originated from 40 cities in 10 provinces in China, with integrated antenatal and postnatal management rate reaching 94.0% (126/134) and a high accuracy rate (99.3%) of prenatal primary diagnosis. The median period of follow-up was 1.6 [interquartile range (IQR) 0.1–4.3] years. There were 3 (2.2%) postnatal deaths, 118 (88.1%) patients undergoing arterial switch operation (ASO), 3 (2.2%) undergoing Rastelli operations and 5 (3.7%) doing stage operations. Of 118 patients receiving ASO, the major morbidity occurred in 64 patients (54.2%), and right ventricular outflow tract obstruction (RVOTO) in 31 (26.3%). In the multivariate logistic analysis, gestational ages at birth (OR = 0.953, 95% CI 0.910–0.991; *p* = 0.025) and cardiopulmonary bypass (CPB) time (OR = 1.010, 95% CI 1.000–1.030; *p* = 0.038) were identified as independent risk factors associated with major morbidity. In the Cox multivariate analysis, aortic cross-clamping time (HR = 1.030, 95% CI 1.000–1.050; *p* = 0.017) was identified as independent risk factor associated with RVOTO.

**Conclusion:**

Earlier gestational ages at birth and longer CPB time are significantly associated with increased morbidity. Integrated prenatal and postnatal management is recommended for patients with prenatal diagnosis of TGA.

**Supplementary Information:**

The online version contains supplementary material available at 10.1186/s13052-024-01730-w.

## Introduction

Transposition of the great arteries (TGA) is the most common cyanotic congenital heart disease (CHD) in neonates, approximately accounting for 0.2/1000 of live births [1, 2]. This kind of ductus arteriosus-dependent congenital heart disease requires an immediate neonatal management to avoid hypoxemia [3]. Without surgical treatment, the natural history was extremely poor; 30% died within days, 50% within 1 month, and 90% within 1 year of birth [4]. Arterial switch operation (ASO), capable of restoring normal hemodynamics by interchanging the position of the two major vessels and grafting coronary, is currently the preferred choice for anatomical correction of TGA. Due to its technically high difficulty, it is often only available at large pediatric cardiology reference centers. Most patients with TGA with intact ventricular septum (TGA/IVS) undergo operation within 3 weeks [1]. Based on major morbidity and healthcare costs, it was suggested that the third day after birth seems to be the best time for ASO [5].

TGA is currently one of the most commonly misdiagnosed CHD in utero. The detection rates of prenatal diagnosis were reported to be fairly low, ranging from 8%-42% [6]. Due to the lack of prenatal diagnosis, patients are often born outside of tertiary-care heart centers and cannot be observed to undergo surgical treatment in time after birth, leading to potentially avoidable death or multi-organ damage [7]. It has been reported that prenatal diagnosis of TGA may allow to reduce early mortality [6, 7]. Even in low-resource areas, fetuses with TGA/IVS benefited from prenatal diagnosis and planned peripartum care [8]. So integrated prenatal and postnatal management is imperative for those with TGA to receive proper perinatal care and prompt surgical treatment. There have been ongoing studies focusing on the preoperative management of TGA patients [5, 9]; however, the comprehensive life-cycle management of these individuals from the fetal stage through postoperative follow-up until death is ill-defined. Therefore, this study aimed to review prenatal diagnosis, associated abnormalities, and mid-term postnatal outcomes of TGA fetuses and investigate the integrated prenatal and postnatal management for TGA neonates.

## Materials and methods

### Study population

This retrospective cohort study included 134 infants prenatally diagnosed with TGA by fetal echocardiography between January 2009 and December 2022 at Guangdong Provincial People’s Hospital, China. The main prenatal diagnoses in this study included: TGA/IVS, TGA with ventricular septal defect (TGA/VSD) with or without arch abnormality, and TGA/VSD with or without left ventricular outflow tract obstruction (LVOTO). Fetuses with severe combined cardiac anomalies such as single ventricle, hypoplastic left heart syndrome, and complete atrioventricular septal defect were excluded, as were fetuses unable to follow the postnatal outcome, whose family chose induced abortion. All data were collected from hospital and outpatient records and completed in May 2023.

### Integrated prenatal and postnatal management strategy

Since 2002, the Chinese Government has established a hierarchical prevention and treatment network for CHD, which included screening in certified primary obstetric medical institutions, diagnosis of CHD subtypes in provincial or municipal tertiary maternal and child health institutions, and specialist consultation and treatment in regional or national clinical research centers [10]. Therefore, those fetuses with abnormalities on prenatal cardiac screening were progressively referred to our center. Then, the fetal echocardiography was performed by pediatric cardiologists in all prenatally diagnosed TGA with a standardized segmental approach. Multidisciplinary team composed of the cardiologists, the cardiac surgeon, and the obstetrician would be formed to provide professional counseling. If patients’ families decided to continue with the pregnancy, they would be enrolled in the procedure that was called integrated prenatal and postnatal management. The pregnant woman was suggested to give birth in the heart center. After birth, the neonates were admitted to the neonatal intensive care unit (NICU) for further treatment and care, and the cardiovascular pediatricians performed the postnatal echocardiography as soon as possible to confirm the diagnosis.

The neonatologists, together with cardiovascular pediatricians and pediatric surgeons, jointly formulated a diagnosis and treatment plan for each neonate and evaluated the use of prostaglandin E. This was based on the infant’s extent of the interatrial communication, oxygen saturation and the outcome of blood gas analysis. If necessary, the infant was given mechanical ventilation, inotropic support and received timely surgical treatment. After the operation, the patient was admitted to the intensive care unit for postoperative recovery, achieving the effect of integrated diagnosis and treatment performed before labor, during delivery, and after birth. Following the discharge from the hospital, the patient would be followed up in the outpatient clinic for a long period, thus realizing the management of the whole lifetime.

### Fetal echocardiography

**Fig. 1:**
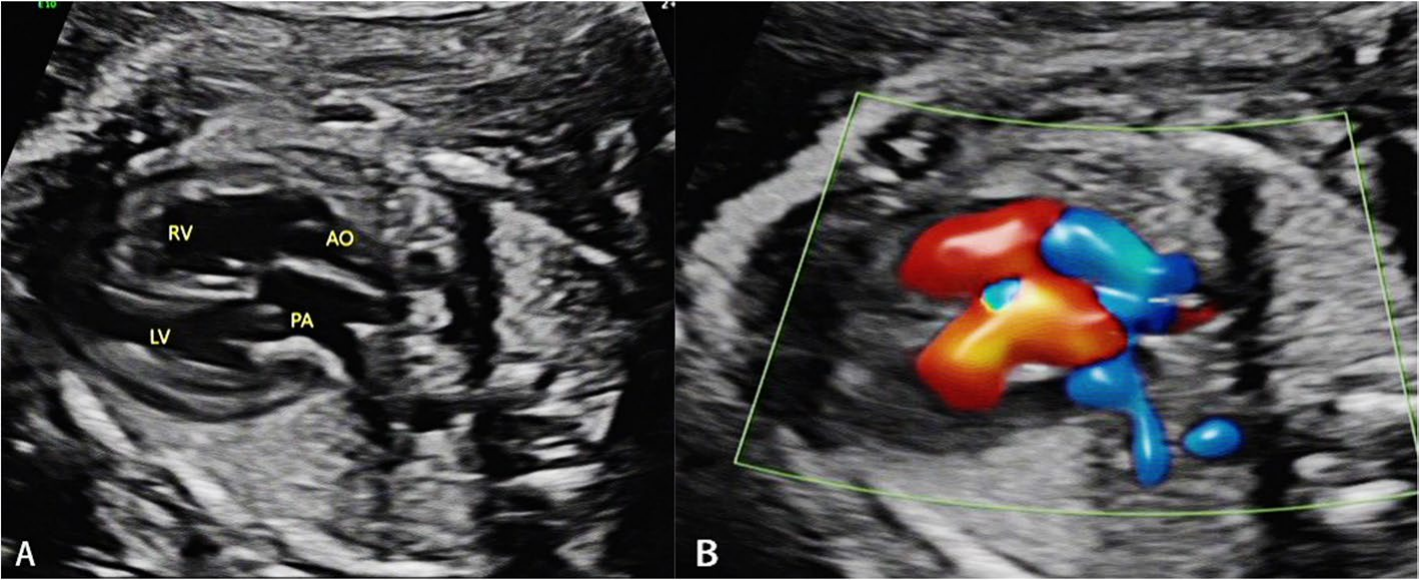
Inconsistent ventricle-vessel connections and the parallelism of the great arteries in two ventricular outflow tracts of TGA. **A** 2-dimensional mapping; **B** Doppler color flow mapping. RV, right ventricle; LV, left ventricle; AO, aorta; PA, pulmonary artery

**Fig. 2:**
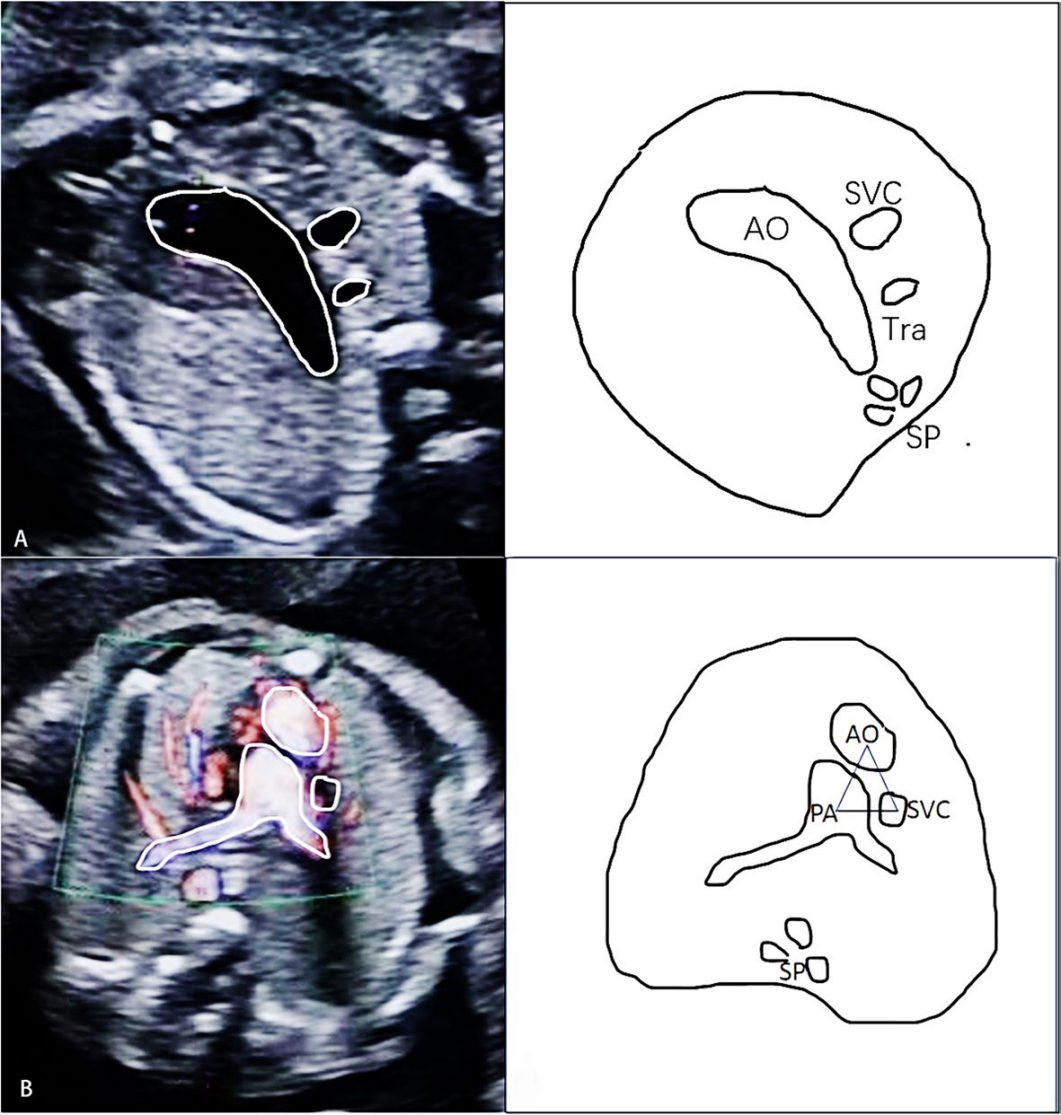
The unusual arrangement of the major vessels. **A** Two vessels are found in the 3-vessel and trachea view because of an anteriorly displaced aorta in TGA; **B** Abnormal alignment of the major vessels are shown as triangle configuration on the 3-vessel view at the level of the pulmonary bifurcation. AO, aorta; SVC, superior vena cava; Tra, trachea; SP, spine

According to the American Society of Fetal Echocardiography guidelines [11], the standard fetal echocardiographic examinations and measurements were performed by experienced pediatric cardiologists, and all complete written reports of the echocardiogram were retained. The fetal heart was examined by using the segmental analysis method, which sequentially examined the position of the heart in relation to the internal organs, the vein-atrium connection, the atrioventricular connection, and the ventricle-vessel connection. It was important to observe the function of the atrioventricular and semilunar valves, the integrity of the interventricular septum, the development of the main pulmonary artery and the branch pulmonary arteries, and the continuity of the aortic arch in the group. The main signs of prenatal diagnosis of TGA were consistent atrioventricular connections and inconsistent ventricular and great vessel connections. Outflow tract views showed that the aorta (AO) arose from the right ventricle and presented as a long vessel, while the pulmonary artery (PA) originated from the left ventricle which appeared as a bifurcation in shape, with the parallelism of the great arteries (shown in Fig. 1). Besides, three vessel view and three-vessel and tracheal view showed an unusual arrangement of the vessels (shown in Fig. 2), which should have shown the normal PA, AO and superior vena cava (SVC) in turn.

### Surgical procedure

ASO was a major surgical strategy for TGA correction. First, a median sternotomy was performed, followed by full flow moderate hypothermic cardiopulmonary bypass (CPB) and aortic cross-clamping (ACC). The right atrium was opened to inspect intracardiac structures. The coronary buttons were trimmed and attached to the pulmonary root after transecting the aorta. With the Lecompte maneuver, the pulmonary artery was located anteriorly. Then the distal aorta was anastomosed to the neoaortic root, and the neopulmonary root was reconstructed by a single autologous pericardial patch and re-attached to the pulmonary artery branch. Coronary artery anatomy was divided into two categories: (i) usual type: 1LCx-2R and 1L-2RCx and (ii) non-usual type: other than (i) [12]. The presence of coronary artery looping pattern, the intramural coronary artery and other abnormalities were also investigated.

### Follow-up

Patients returned for follow-up appointments at least every 6 months during the first 3 years and annually thereafter until death. The primary endpoint was major morbidity, which was defined as cardiac arrest, extracorporeal membrane oxygenation, delayed sternal closure, other reoperation before discharge, diaphragmatic paralysis/paresis, systemic infection (such as sepsis and fungemia), necrotizing enterocolitis, seizure, stroke on MRI with associated clinical findings, or readmission within 30 days [5]. The time of the last follow-up was 31 May 2023, with a maximum follow-up of more than twelve years. Mortality was defined as early (within 30 days) or late. With continuous Doppler on echocardiography measuring the maximum velocities across the right ventricular outflow tract and pulmonary artery, right ventricular outflow tract obstruction (RVOTO) was defined as measurable gradients > 30 mmHg across the stenosis area [13].

### Statistical analysis

R software (version 4.2.4, R Foundation for Statistical Computing, Vienna, Austria) was used for all statistical analyses and data visualization. Student’s t-test was used for normally distributed data, whereas the Mann–Whitney U-test for non-normally distributed data and the ${x}^{2}$ test for categorical variables. The freedom from death after ASO and freedom from cardiac reoperation after discharge were estimated using Kaplan–Meier curve. Significant variables in univariate analysis (*p* < 0.05) were further tested in multivariate analyses using the Logistic and Cox regression analyses. A 2-tailed *p*-value of < 0.05 was considered statistically significant.

## Results

### Integrated management for total patients

**Fig. 3:**
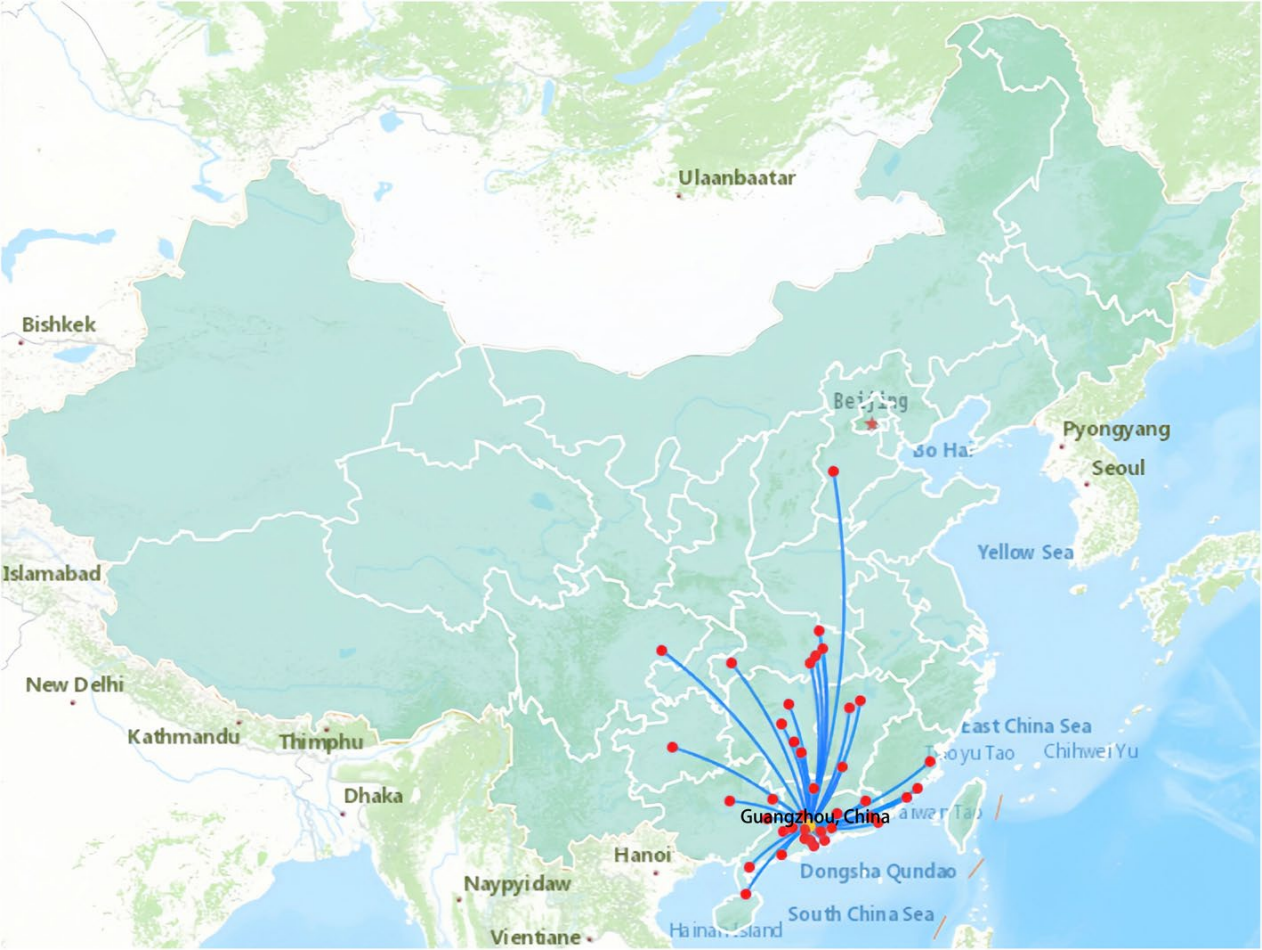
Flow map of the group with the prenatal diagnosis of TGA

**Fig. 4:**
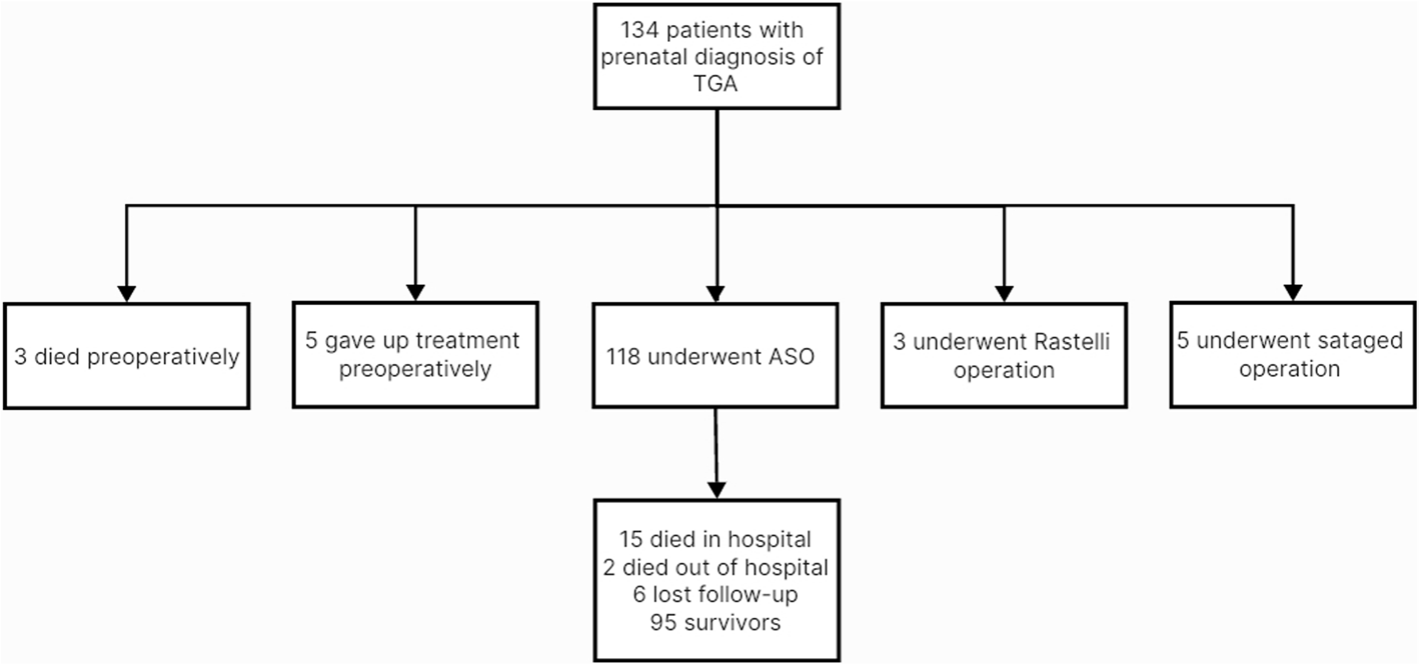
Flowchart of the study population in the group with prenatal diagnosis of TGA. TGA: transposition of the great arteries; ASO: arterial switch operations

Among the 134 TGA patients (11.2% females) during the present study, the population originated from 40 cities in 10 provinces in China, north to Shijiazhuang Hebei, south to Haikou Hainan, east to Fuzhou Fujian and west to Nanchong Sichuan (shown in Fig. 3). The main sources were Guangzhou (34 cases), Foshan (11 cases), and Dongguan (11 cases) in Guangdong Province. All fetuses were alive at delivery. As an important referral center for prenatal diagnosis in South China, our center achieved an integrated management rate of 94.0% (126/134). 7 of the rest were transferred from other hospitals within seven days after birth, and 1 patient arrived at 52 days old due to family custom. The intention-to-treat survival rate was 96.3% (129/134), while 5 cases declined the treatment preoperatively. There were 3 (2.2%) postnatal deaths, 118 (88.1%) patients undergoing ASO, 3 (2.2%) undergoing Rastelli operations and 5 (3.7%) doing stage operations (shown in Fig. 4). The median follow-up period for the entire cohort was 1.57 years [interquartile range (IQR) 0.07–4.28], with a maximum follow-up of 12.2 years.

### Prenatal and postnatal echocardiography of total patients

Of the entire cohort, all cases were born and had at least one postnatal echocardiography. The mean gestational age at first diagnosis was 27.7 weeks (Range, 19–40). Anatomic structure at surgery was considered the gold standard to confirm the accuracy of ultrasonic diagnosis, but if there were no surgeries performed, a definitive diagnosis was based on postnatal imaging. Among 134 patients, there were 126 cases (94.0%) of TGA, 7 cases (5.2%) of Taussig Bing anomaly (TBA), and 1 case of double outlet of right ventricle with VSD far away from the aortic and pulmonary valves. Additionally, 55 (41.0%) patients had VSD, 10 (7.5%) had arch abnormality and 9 (6.7%) had LVOTO.

The correspondence between prenatal and postnatal ultrasound diagnosis and surgical diagnosis was shown in Table 1. In terms of the accuracy of primary diagnosis, the difference between the two diagnostic modalities was not statistically significant (*p* = 1.000). The prenatal primary diagnosis was correct in 99.3% of the cases. However, if the integrity of the interventricular septum, obstruction of the ventricular outflow tract and assessment of aortic arch continuity were taken into account, the accuracy of prenatal ultrasound decreased to 60.4%, and that of postnatal ultrasound was 88.8%, with a significant difference (*p* < 0.001).

**Table 1:** Comparison of the diagnostic accuracy of prenatal and postnatal ultrasound

	Surgical diagnosis		Total	Accuracy (%)	*P*-value
	Consistency	Inconsistency			
Prenatal ECHO primary diagnosis	133	1	134	99.3	1.000
Postnatal ECHO primary diagnosis	132	2	134	98.5	
Total	265	3	268	98.9	
Prenatal ECHO primary and secondary diagnosis	81	53	134	60.4	0.000
Postnatal ECHO primary and secondary diagnosis	119	15	134	88.8	
Total	200	68	268	74.6	

*Abbreviation*: *ECHO* Echocardiography

### Characteristics of patients with ASO

In this study, a total of 118 patients underwent ASO. Their baseline characteristics are shown in Table 2. All cases were prenatally diagnosed with TGA, while in 22 both TGA/VSD and TBA have been suggested, as these two diseases are difficult to distinguish in utero. Therefore, 46 cases were prenatally diagnosed with TGA/IVS, 72 with TGA/VSD or TBA, 19 with arch abnormality and 5 with LVOTO. The sizes of VSD, ductus arteriosus (DA), and foramen ovale (FO) were mainly focused on in TGA fetuses. The median VSD/AO ratio in prenatal echocardiography was 0.6 (IQR 0.0–0.9), DA/AO ratio 0.6 (IQR 0.5–0.8) and FO/AO ratio 1.1 (IQR 0.9–1.5).

**Table 2:** Prenatal and postnatal clinical characteristics of patients receiving ASO

Characteristic	Overall (*n* = 118)
**Pre-delivery**	
Prenatal diagnosis, n (%)	
TGA/IVS	46 (39)
TGA/VSD or TBA	72 (61)
Other cardiac anomalies, n (%)	
Arch abnormality	19 (16)
LVOTO	5 (4)
Parameters of prenatal Echo, median [IQR]	
VSD/AO ratio	0.6 (0.0–0.9)
DA/AO ratio	0.6 (0.5–0.8)
FO/AO ratio	1.1 (0.9–1.5)
**After delivery**	
Male, n (%)	105 (89)
Gestational age (weeks), median [IQR]	38.6 (37.1–39.3)
Term infant, n (%)	97 (82)
Weight < 2500g, n (%)	15 (13)
Blood-oxygen saturation (%)	75.0 (60.0–85.0)
PH in blood gas analysis, median [IQR]	7.31 (7.26–7.36)
Lactate in blood gas analysis (mmol/L), median [IQR]	2.5 (1.8–3.9)
Prostaglandin E1-dependent before surgery, n (%)	68 (58)
Parameters of Postnatal Echo	
VSD/AO ratio, median [IQR]	0.0 (0.0–0.4)
Shunt flow volume of atrial septal (mm), median [IQR]	4.5 (2.7–6.1)
PDA/AO ratio, median [IQR]	0.5 (0.4–0.6)
**Intraoperative variables**	
Emergency operation, n (%)	39 (33)
Age at ASO (d), median [IQR]	8.0 (4.8–11.0)
CPB time (min), median [IQR]	185.0 (158.5–219.0)
ACC time (min), median [IQR]	111.0 (93.0–126.0)
Surgical procedures, n (%)	
VSD repair	41 (35)
Concomitant arch repair	7 (6)
Coronary artery anatomy, n (%)	
Usual (1LCx-2R, 1L2RCx)	87 (74)
Other (non-usual)	31 (26)
Coronary anomaly, n (%)	
Looping pattern	20 (17)
Intramural coronary artery	7 (6)
Coronaries from a single ostium	6 (5)

*Abbreviations*: *ASO* Arterial switch operation, *TGA/IVS* Transposition of the great arteries with intact ventricular septum, *TGA/VSD* Transposition of the great arteries with ventricular septal defect, *TBA* Taussig-Bing anomaly, *LVOTO* Left ventricular outflow tract obstruction, *IQR* Interquartile range, *ECHO* Echocardiography, *VSD* Ventricular septal defect, *AO* Aorta, *DA* Ductus arteriosus, *FO* Foramen ovale, *PDA* Patent ductus arteriosus, *CPB* Cardiopulmonary bypass, *ACC* Aortic cross-clamping, *Cx* Circumflex artery, *L* Left coronary artery, *R* Right coronary artery, *1* Sinus 1, *2* Sinus 2

With 97 (82%) term infants in these 118 patients, the median gestational age at birth was 38.6 (IQR 37.1–39.3) weeks. There was a male predominance (89% males). Fifteen (15/118) patients had a birth weight of less than 2500g. On admission to the NICU, the median oxygen saturation fluctuated at 75.0 (IQR 60.0–85.0) %. Routine blood gas analysis was performed with a median pH of 7.31 (IQR 7.26–7.36) and a median lactate concentration of 2.5 (IQR 1.8–3.9) mmol/L. Before ASO, 68 neonates were treated with prostaglandin E1. Balloon atrial septostomy was not routinely used in our center, so no patient in this study underwent this treatment.

The majority of patients performed their first echocardiogram within 24 h of birth. The median VSD/AO ratio was 0.0 (IQR 0.0–0.4), while patent ductus arteriosus (PDA)/AO diameter ratio was 0.5 (0.4–0.6). Atrial septal defect (ASD) and/or patent foramen ovale (PFO) were observed in all infants. The size of ASD and that of PFO were combined in this study to precisely describe the shunt flow volume of the atrial septal. The median volume was 4.5 (IQR 2.7–6.1) mm.

The majority of them received surgical correction within two weeks (median age: 8 days). 39 infants underwent emergency surgeries as the patients’ condition required. Median CPB time and ACC times were 185.0 (IQR 158.5–219.0) min and 111 (IQR 93.0–126.0) min, respectively. Forty-one infants (35%) underwent VSD closure and 7 patients received concomitant arch repair. Coronary arteries with usual origin and alignment, 1LCx2R and 1L2RCx, were found in 87 (74%) cases. The looping pattern was found in 20 cases (17%), of which 8 cases showed the right coronary artery looped anterior to the aorta, and the left coronary artery looped behind the pulmonary artery. Seven cases (6%) had intramural patterns and six cases (5%) had both left and right coronary arising from a single ostium. More anatomic details of coronary artery were shown in Table 2.

### Follow-up of patients with ASO

The major morbidity occurred in 64 patients, including delayed sternal closure (*n* = 39), reoperation before discharge (*n* = 27), cardiac arrest (*n* = 16), diaphragmatic paralysis/paresis (*n* = 14), extracorporeal membrane oxygenation (*n* = 8), systemic infection (*n* = 7) and necrotizing enterocolitis (*n* = 5) (Table 3). More clinical data between the group of major morbidity and the rest were shown in Supplementary material Table S1. In univariable analyses, VSD/AO ratio in prenatal echocardiography, VSD/AO ratio in postnatal echocardiography, the shunt flow volume of atrial septal in postnatal echocardiography, gestational ages at birth, CPB and ACC time were significantly associated with the major outcome (Table 4). In the multivariate analysis, gestational ages at birth (OR = 0.953, 95% CI 0.910–0.991; *p* = 0.025) and CPB time (OR = 1.010, 95% CI 1.000–1.030; *p* = 0.038) were risk factors for major morbidity.

**Table 3:** Major postoperative morbidity [5]

Major morbidity	N (%)
Arrest	16 (13.6%)
Extracorporeal membrane oxygenation	8 (6.8%)
Delayed sternal closure	39 (33.1%)
Other reoperation before discharge	27 (22.9%)
Diaphragmatic paralysis/paresis	14 (11.9%)
Systemic infection	7 (5.9%)
Necrotizing enterocolitis	5 (4.2%)
Seizure	0 (0.0%)
Stroke by MRI with associated clinical findings	0 (0.0%)
Readmission within 30 days	0 (0.0%)

**Table 4:** Univariate and multivariate analyses of risk factors for major morbidity

Characteristic	Univariate analysis		Multivariate analysis	
	OR (95%CI)	*P*-value	OR (95%CI)	*P*-value
VSD/AO in prenatal ECHO	2.158 (1.010–4.448)	0.030		
VSD/AO in postnatal ECHO	3.158 (1.095–10.008)	0.040		
The shunt flow volume of atrial septal in postnatal ECHO	0.838 (0.697–0.993)	0.048		
Gestational ages at birth	0.957 (0.920–0.992)	0.022	0.953(0.910–0.991)	0.025
CPB time	1.013 (1.005–1.022)	0.003	1.010(1.000–1.030)	0.038
ACC time	1.016(1.002–1.032)	0.033		

*Abbreviations*: *VSD* Ventricular septal defect, *AO* Aorta, *ECHO* Echocardiography, *CPB* Cardiopulmonary bypass, *ACC* Aortic cross-clamping

**Fig. 5:**
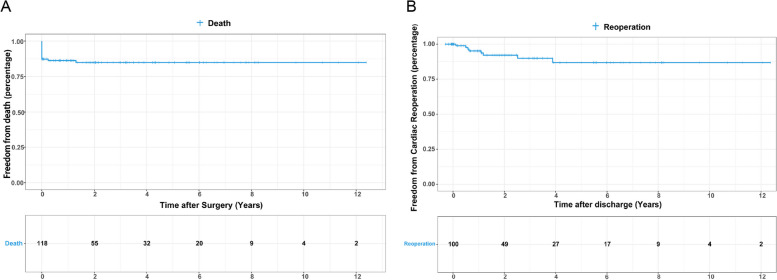
Kaplan–Meier estimates of overall survival after the ASO (**A**) and freedom from cardiac reoperation after discharge (**B**)

The overall mortality rate of postoperative patients with ASO was 14.4% (17/118). Hospital mortality was 12.7% (15 patients). Two died late during follow-up. Kaplan–Meier plot showing survival after ASO is shown in Fig. 5A. The estimated survival rate was 87.3% at 1 month, 86.3% at 1 year, and 84.9% at 5 and 10 years. A vast majority of postoperative deaths occurred in the early postoperative period, and the survival rate remained stable in the later long-term follow-up. The main causes of the early death were low cardiac output syndrome and multiple organ failure (*n* = 10). Three died of heart failure, one of septic shock and one of superior vena caval obstruction syndrome. Among the late deaths, both two had cardiac shock.

With median follow-up after discharge of 1.8 (IQR 0.2–4.3) years, cardiac reoperations during follow-up occurred in 8 patients. Seven of them developed vascular anastomotic stenosis in the neopulmonary artery or neoaorta, and one underwent aortic valvuloplasty for severe aortic regurgitation. Freedom from reoperation was 95.2%, 86.8%, and 86.8% at 1, 5, and 10 years, respectively (shown in Fig. 5B).

26.3 percent (*n* = 31) experienced RVOTO after surgery. RVOTO existed persistently in 17 patients, while the rest of them had RVOTO at the early stage after the operation but had a better evolution during follow-up. DA/AO ratio in prenatal echocardiography, pulmonary valve (PV) velocity in postnatal echocardiography, term infant, ACC time and hemorrhage volume during ASO were univariable risk factors for the RVOTO after ASO (shown in Table 5). Independent risk factors were DA/AO ratio in prenatal echocardiography (HR = 16.800, 95% CI 1.230–230.000; *p* = 0.034) and ACC time (HR = 1.030, 95% CI 1.000–1.050; *p* = 0.017).

**Table 5:** Univariate and multivariate analyses of risk factor RVOTO in patients after ASO

Characteristic	Univariate analysis		Multivariate analysis	
	HR (95%CI)	*P*-value	HR (95%CI)	*P*-value
DA/AO in prenatal ECHO	12.626 (2.562—62.233)	0.002	16.800 (1.230–230.000)	0.034
PV velocity in postnatal ECHO	3.671 (1.507—8.945)	0.004		
Term infant	2.502 (1.169—5.353)	0.018		
ACC time	1.015 (1.004—1.027)	0.010	1.030 (1.000–1.050)	0.017
Hemorrhage volume during ASO	1.004 (1.001—1.008)	0.008		

*Abbreviations*: *DA* Ductus arteriosus, *AO* Aorta, *ECHO* Echocardiography, *PV* Pulmonary valve, *ACC* Aortic cross-clamping, *ASO* Arterial switch operation

## Discussion

To the best of our knowledge, this is the largest cohort of fetuses prenatally diagnosed with TGA in a single center. In this 13-year observational study, we evaluated the surgical outcome and long-term prognosis of infants with TGA who received integrated prenatal and postnatal management. Benefiting from the three-level prevention and treatment network for CHD in China [10], our center, a major referral center, provided specialist consultation and treatment services for CHD in South China. The prenatal diagnosis coverage of TGA reached 40 cities in 10 provinces. Similarly, a regional network for prenatal diagnosis and referral of patients with critical CHD was initiated in Kochi, Kerala, India, serving a population of 35 million people [8]. Such a network enabled planned peripartum care for TGA infants in specialist centers and improved neonatal survival, especially in low-resource setting. Even in high-income countries, like Italy, the differences in health outcomes were found among different population group for social and orographic reasons. Adequate resources and intervention strategies should be addressed to the management of prematurity and pathology like CHD [14]. The network for CHD prevention and treatment may be beneficial to the area with uneven resource distribution in rich countries.

Among the 134 patients prenatally diagnosed with TGA during the study period, we achieved a high accuracy rate of prenatal primary diagnosis (99.3%), comparable to the foreign diagnostic level (93.3% [15]-96.1% [16]). The preoperative mortality rate was low (2.2%), compared with 4–6% reported in other countries [7]. Rashkind procedure is not a routine for neonates with TGA in our hospital because the related balloon has not been approved by National Medical Products Administration in China, which differs from the postnatal management of TGA in foreign centers. In addition, the development of pediatrics contributed to a progressive improvement in the quality of perinatal management, children’s care and surgical technique, and in the meantime to the decrease of neonatal and infant mortality rates [17]. Due to the integrated management of patients, most of these infants were born in our center and we could use prostaglandin E in time to keep the ductus arteriosus open so their blood oxygen saturation could maintain a satisfactory level. Rarely, emergency surgery would be performed in time to correct TGA in the context of severe hypoxemia refractory to medication.

In terms of the accuracy of prenatal and postnatal diagnosis, we observed that if the primary diagnosis was the key to judgment, the accuracy of prenatal ultrasound diagnosis in this study reached 99.3%, which was not significantly different from postnatal ultrasound diagnosis. However, when the integrity of ventricular septum, patency of the outflow tract and continuity of the aortic arch were taken into account, the accuracy of prenatal ultrasound diagnosis decreased to 60.4%, which was significantly different from that of postnatal ultrasound. Prenatal ultrasound lacked the advantage of a definitive secondary diagnosis, mainly because pulmonary stenosis and aortic constriction may develop further in utero, making the degree of stenosis difficult to predict [15]. Because of the small difference in pressures between the right and left ventricles during fetal circulation, VSD is difficult to diagnose by ultrasound. Moreover, the presence, size, and location of the VSD may be affected by many factors such as acoustic restriction, quality of the image obtained and gestational age at the time of scanning. There were 33 cases diagnosed with VSD prenatally but without VSD postnatally. For prenatal counseling of such patients with TGA/VSD, pregnant women should be advised to stay near a cardiac center with neonatal surgical expertise. The patients should receive integrated antenatal and postnatal management to avoid severe hypoxemia at birth and the risk of death during transport. Therefore, among the CHD prevention strategies, prenatal ultrasound evaluations may be crucial for the proper management of patients. TGA is likely to have a polygenic inheritance and related to a susceptibility locus on chromosome 3p14.3 near *WNT5A* [18]. In the context of complex congenital cardiopathies, early genetic diagnosis may also contribute to a better management of patients in specialized centers, individualized follow-up to prevent future comorbidities, and to provide more precise prognostic evaluations and genetic counselling to the involved families, including communication of recurrence risk for later pregnancies [19–22].

We also identified some factors that might affect the mortality and morbidity of TGA. In this retrospective study, we found that older gestational age at birth was associated with decreased morbidity. This finding is consistent with others’ results like Hautala et al. [23] and Boos et al. [24]. Preterm birth is often associated with low birth weight [25], especially in children with TGA, and many large multi-institutional studies have shown increased mortality in infants weighing < 2500g after ASO surgery [13]. Among the 21 preterm infants in our study, the median weight was 2430g (range 1400–3020) g, with 19% (4/21) death rate. Preterm infants with TGA may combine with co-morbidities typical for prematurity leading to increased mortality, including respiratory distress syndrome, bronchopulmonary dysplasia and necrotizing enterocolitis [24]. Unless there are obstetric indications, it is preferable to deliver at 39 to 40 weeks, and children delivered at term have better long-term neurodevelopmental outcomes [2]. A longer CPB time is a risk factor for major morbidity, which has been confirmed in many studies [26, 27].

RVOTO is the most common complication after ASO, including pulmonary artery and branch pulmonary artery stenosis, which often requires postoperative reoperation [28]. The causes of postoperative branch pulmonary artery stenosis are unclear and may involve different mechanisms. One of the possible causes is that the fixed pulmonary arteries at the hilum of lung, after intraoperative LeCompte maneuver in ASO, may limit the activity of the branch pulmonary arteries [28]. This results in stretching or even flattening of the left and right pulmonary arteries. Another reason may be the decrease in blood flow and pressure in the branch pulmonary arteries after ASO, resulting in a decrease in the internal diameter of the right and left pulmonary arteries [29]. Our findings show that those who had a higher DA/AO ratio in the prenatal echocardiography and longer ACC time were more likely to have recurrent RVOTO. The smaller aortic valve and aortic isthmus and larger DA in fetuses are associated with coarctation of the aorta in previous study [30]. Cleuziou et al. [31] found that RVOTO after operations was more frequently observed in those with TBA and aortic arch anomalies and those with unusual coronary patterns requiring extensive and complex translocation. All these morphological characteristics accordingly require longer ACC time. ACC time was also regarded as an independent risk factor in reintervention mostly of pulmonary stenosis [32], which was consistent with our study.

## Study limitations

Our study has some limitations that should be acknowledged. First, this was a retrospective study with a relatively limited sample size and a single-center design, which might limit the generalizability of our findings. Second, we did not measure the pulmonary artery pressure and resistance of all patients in ASO, which might affect the development and progression of RVOTO. Therefore, further studies with larger samples, multicenter collaborations, and longer-term follow-ups are needed to validate and extend our results.

## Conclusion

For those with prenatal diagnosis of TGA, integrated prenatal and postnatal management is recommended. High prenatal diagnostic accuracy facilitates guidance for perinatal management of infants with TGA. Earlier gestational ages at birth and longer CPB time were independent risk factors for increased morbidity.

### Supplementary Information

Supplementary Material 1

## Data Availability

The raw data supporting the conclusions of this article will be made available by the authors, without undue reservation.

## Abbreviations

CHD: Congenital heart disease
TGA: Transposition of the great arteries
IQR: Interquartile range
ASO: Arterial switch operation
RVOTO: Right ventricular outflow tract obstruction
IVS: Intact ventricular septum
VSD: Ventricular septal defect
LVOTO: Left ventricular outflow tract obstruction
NICU: Neonatal intensive care unit
PA: Pulmonary artery
SVC: Superior vena cava
CPB: Cardiopulmonary bypass
ACC: Aortic cross-clamping
TBA: Taussig Bing anomaly
DA: Ductus arteriosus
AO: Aorta
FO: Foramen ovale
PDA: Patent ductus arteriosus
ASD: Atrial septal defect
PFO: Patent foramen ovale
