# Supplementary material for: Integrated prenatal and postnatal management for neonates with transposition of the great arteries: thirteen-year experience at a single center
Source: Ital J Pediatr. 2024 Aug 22;50:153. doi: 10.1186/s13052-024-01730-w (PMC11340064; doi:10.1186/s13052-024-01730-w)
Supplement: Supplementary file 1 — Supplementary Material 1 [file 13052_2024_1730_MOESM1_ESM.docx]

**Supplementary material**

**Table S1**. Predictors of Morbidity in Univariable Analyses

|  | Survived | Major morbidity | *p*-Value |
| --- | --- | --- | --- |
| Prenatal diagnosis, n (%) |  |  |  |
| TGA/IVS | 26 (48.1) | 20 (31.3) | 0.062 |
| TGA/VSD or TBA | 28 (51.9) | 44 (68.8) |  |
| Additional cardiac anomalies, n (%) | | | |
| Arch abnormality | 8 (14.8) | 11 (17.2) | 0.727 |
| LVOTO | 3 (5.6) | 2 (3.1) | 0.519 |
| Prenatal ECHO |  |  |  |
| VSD/AO | 0.3 (0.0-0.8) | 0.7 (0.0-1.1) | 0.030 |
| DA/AO | 0.6 (0.5-0.8) | 0.7 (0.5-0.7) | 0.403 |
| FO/AO | 1.1 (0.9-1.5) | 1.1 (0.9-1.3) | 0.683 |
| Gestational age (weeks) | 39.0 (38.0-39.6) | 38.3(36.9-39.3) | 0.022 |
| Term infant | 49 (90.7) | 48 (75.0) | 0.032 |
| Male | 48 (88.9) | 57 (89.1) | 0.976 |
| Weight (g) | 3200 (2900-3300) | 3000 (2700-3300) | 0.104 |
| Postnatal ECHO |  |  |  |
| VSD/AO | 0.0 (0.0-0.4) | 0.1 (0.0-0.7) | 0.040 |
| The shunt flow volume of atrial septal | 4.8 (3.2-6.4) | 4.0 (2.6-5.9) | 0.048 |
| PDA/AO | 0.5 (0.4-0.5) | 0.5 (0.4-0.6) | 0.265 |
| Blood-oxygen saturation, % | 75.0 (61.5-83.5) | 75.5 (58.5-85.0) | 0.996 |
| Systolic blood pressure of right leg (mmHg) | 68.0 (62.0-74.3) | 67.0 (60.0-71.8) | 0.141 |
| Diastolic blood pressure of right leg (mmHg) | 32.0 (28.0-37.3) | 30.0 (25.0-33.8) | 0.040 |
| PH | 7.32 (7.26-7.36) | 7.29 (7.26-7.36) | 0.509 |
| Lactate | 2.1 (1.7-4.4) | 2.7 (1.9-3.9) | 0.836 |
| Age at ASO (d) | 8.0 (6.0-10.0) | 8.0 (3-12.8) | 0.463 |
| CPB time (min) | 174.5 (147.8-193.3) | 191.5 (170.3-238.8) | 0.003 |
| ACC time (min) | 100.5 (86.8-118.5) | 115.0 (99.0-126.0) | 0.033 |
| Ultrafiltration in CPB (ml) | 300.0 (150.0-520.0) | 350.0 (200.0-600.0) | 0.156 |
| Hemorrhage volume | 80.0 (50.0-105.0) | 100.0 (50.0-200.0) | 0.057 |
| Concomitant arch repair | 3 (5.6) | 4 (6.3) | 0.874 |

Abbreviations as in Table 2
